# How microbiomes can help inform conservation: landscape characterisation of gut microbiota helps shed light on additional population structure in a specialist folivore

**DOI:** 10.1186/s42523-021-00122-3

**Published:** 2022-01-31

**Authors:** B. L. Littleford-Colquhoun, L. S. Weyrich, K. Hohwieler, R. Cristescu, C. H. Frère

**Affiliations:** 1 Global Change Ecology, School of Science and Engineering, University of the Sunshine Coast, Sippy Downs, QLD 4556 Australia; 2 Department of Ecology, Evolution and Organismal Biology, Brown University, Providence, RI 02912 USA; 3 Department of Anthropology and Huck Institutes of the Life Sciences, The Pennsylvania State University, University Park, PA 16802 USA; 4 School of Biological Sciences, The University of Adelaide, Adelaide, SA 5005 Australia; 5 Institute at Brown for Environment and Society, Brown University, Providence, RI 02912 USA

**Keywords:** Koala, Gut microbiota, Conservation, Specialist species, Dietary specialist, Landscape variation

## Abstract

**Background:**

The koala (*Phascolarctos cinereus*), an iconic yet endangered specialised folivore experiencing widespread decline across Australia, is the focus of many conservation programs. Whilst animal translocation and progressive conservation strategies such as faecal inoculations may be required to bring this species back from the brink of extinction, insight into the variation of host-associated gut microbiota and the factors that shape this variation are fundamental for their success. Despite this, very little is known about the landscape variability and factors affecting koala gut microbial community dynamics. We used large scale field surveys to evaluate the variation and diversity of koala gut microbiotas and compared these diversity patterns to those detected using a population genetics approach. Scat samples were collected from five locations across South East Queensland with microbiota analysed using 16S rRNA gene amplicon sequencing.

**Results:**

Across the landscape koala gut microbial profiles showed large variability, with location having a large effect on bacterial community composition and bacterial diversity. Certain bacteria were found to be significantly differentially abundant amongst locations; koalas from Noosa showed a depletion in two bacterial orders (*Gastranaerophilales* and *Bacteroidales*) which have been shown to provide beneficial properties to their host. Koala gut microbial patterns were also not found to mirror population genetic patterns, a molecular tool often used to design conservation initiatives.

**Conclusions:**

Our data shows that koala gut microbiotas are extremely variable across the landscape, displaying complex micro- and macro- spatial variation. By detecting locations which lack certain bacteria we identified koala populations that may be under threat from future microbial imbalance or dysbiosis. Additionally, the mismatching of gut microbiota and host population genetic patterns exposed important population structure that has previously gone undetected across South East Queensland. Overall, this baseline data highlights the importance of integrating microbiota research into conservation biology in order to guide successful conservation programs such as species translocation and the implementation of faecal inoculations.

**Supplementary Information:**

The online version contains supplementary material available at 10.1186/s42523-021-00122-3.

## Background

Vertebrate species harbour diverse and complex communities of microbes, which are adapted to live in and on the body of their host [1, 2]. The gut microbial community is one of the most influential of these symbiotic communities [3], playing a critical role in a variety of processes which affect host health and fitness, including metabolism, nutrition, immunology, behaviour, morphology and development [4]. However, despite the growing evidence over the last century describing the influence gut microorganisms have on vertebrate species [5], the field of conservation biology has largely concentrated on the macroecological, rather than the microbial world [6–8].

New paradigms within conservation biology are now required given that we have entered the Anthropocene, where human-induced environmental change is the dominant force [9] and current extinction rates are unprecedented in human history [10, 11]. Human impacts on the natural environment are far reaching and are likely to impact not only macroecological but also microbial diversity [12]. Whilst the goals of conservation biology are to maintain biological diversity, ecological integrity, and ecological health [13, 14], these goals are largely centred around taxonomy, ecology, genetics and evolutionary biology [14], meaning that the intricate relationships between host and its microbiome (termed the holobiont) are not often considered [7]. Neglecting to incorporate metagenomic research into conservation biology can lead to the mismanagement of individuals, populations and even species. Once a wild host species becomes locally extinct, it is also possible that its unique symbionts may also perish [15]. Gut microbial studies can not only identify microorganisms important for animal health, survival, and fitness [16], but can also help shape conservation initiatives where animals may be faced with a sudden change in environmental conditions and/or diet, such as animal translocation [17] or reintroduction programmes [7, 18]. Baseline knowledge of host-microbial interactions provides an important first step in determining whether specific gut microbes (or microbial genes) are required to prime the host’s immune system in order to aid resistance to future environmental perturbations and/or pathogens.

The inclusion of microbiome research in conservation may be especially important when considering the effect of perturbations on dietary specialist species, as thee species rely heavily on very specific food types and often require a specific microbial assemblage for effective digestion [7, 19]. The koala (*Phascolarctos cinereus*) is one such specialist species; this species is not only a specialised folivore, feeding primarily on the tree genus *Eucalyptus*, but is also considered vulnerable to extinction across much of its current range [20, 21] with many populations suffering declines of up to 80% in the last two decades [22–24]. The diet of the koala can vary significantly in nutritional quality and toxicity depending on which species of eucalypt are consumed [25], therefore, the selection of appropriate food trees is a critical skill and is thought to be facilitated by the expansion of multiple gene families associated with olfaction and taste receptors [26]. Food choice and the ability to digest and detoxify eucalypt leaves is further influenced by experience, physiology, and the microbiome [17]. Indeed, the koala relies heavily on the microorganisms in their gut for the fermentation of dietary fibre and other refractory materials [27–30], and the detoxification of plant secondary metabolites [25, 31]. Whilst comparative analyses suggest that diet is a major environmental factor contributing to gut microbial variation between mammalian species [32], host-specific factors like co-colonization with enteric parasites and disease [33, 34], and host genetics [35, 36] may also contribute to inter-individual and temporal variations in gut microbial community structure [37]. Koalas first acquire their gut microbiomes by ingesting pap (faecal matter) excreted by the mother around the time of pouch emergence [38]. Pap is extremely concentrated in nutrients and microbes, and is thought to be an essential component for joeys to transition to an adult diet [17, 38].

Whilst an emergent body of conservation research has focussed on the landscape genetics of the koala [26, 39–42], few studies have focussed on the koala microbiome, with those published having concentrated on a single population [17, 43], very few individuals [29], or animals in captivity, zoos or veterinary clinics/animal hospitals [28, 30, 44]. Early culture-based investigations of the koala gut microbiome identified *Streptococcus gallolyticus* and *Lonepinella koalarum* as important bacteria for tannin degradation [45, 46], whilst more recent amplicon sequencing has identified *Bacteroides* and *Ruminococcus* as being important genera for metabolising complex plant compounds into short-chain fatty acids, and bacterial members of the family *Synergistaceae* as being important for the degradation of toxic Eucalyptus plant secondary metabolites [28]. Despite this research, there still remains a lack of fundamental knowledge as to the koala’s ‘natural’ gut microbial variation across the landscape. It is important that we understand this landscape variation, not only because the koala is an important and vulnerable species, but because novel or critical microbial species may be uncovered.

By combining large scale field surveys, the sequencing of the V3–V4 region of the 16S rRNA gene, and SNP genotyping, we were able to evaluate the variation and diversity of koala gut microbiotas and compare these patterns of diversity to those detected using a population genetics approach. We show for the first time that (1) koala gut microbial profiles show large variability across the landscape and (2) that koala landscape microbial patterns do not match landscape population genetic patterns which are often considered when designing koala conservation initiatives.

## Results

**Fig. 1:**
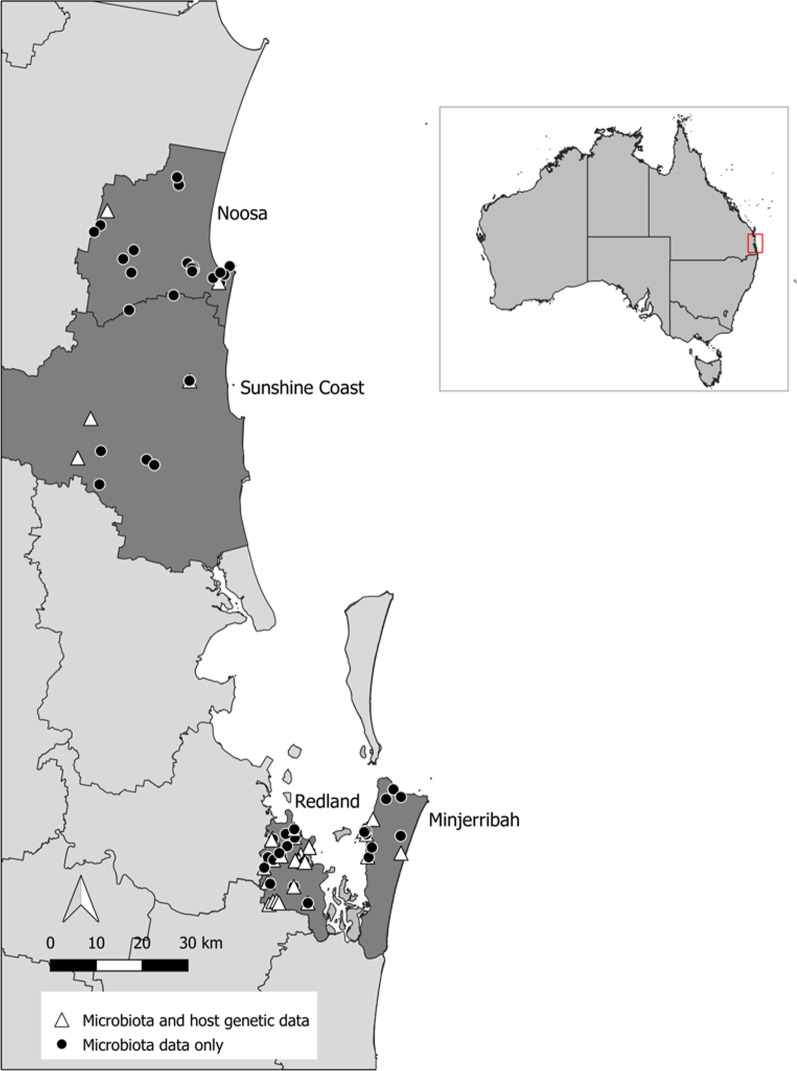
Map showing all locations of scat samples (prior to filtering; *n* = 96) collected across four locations in South East Queensland, Australia (locations highlighted in dark grey with council borders outlined by black lines). White triangles represent samples which yielded microbiota and koala DNA data, black circles represent samples with microbiota data only. Please see Additional file 1: Figure S1 for fine-scale maps outlining the number of overlapping samples in each location

To determine how the gut microbiota of koalas varied across the landscape, we looked at whether there were significant differences in microbial diversity across four locations in SEQ. Location boundaries were defined by local council borders (Fig. 1), and whilst we acknowledge that koalas do not recognise these anthropomorphised borders and may pass between them, koala conservation is often conducted at the local council level, therefore, for this research to be valuable to council conservation strategies, we maintained these council management units within our analyses.

**Fig. 2:**
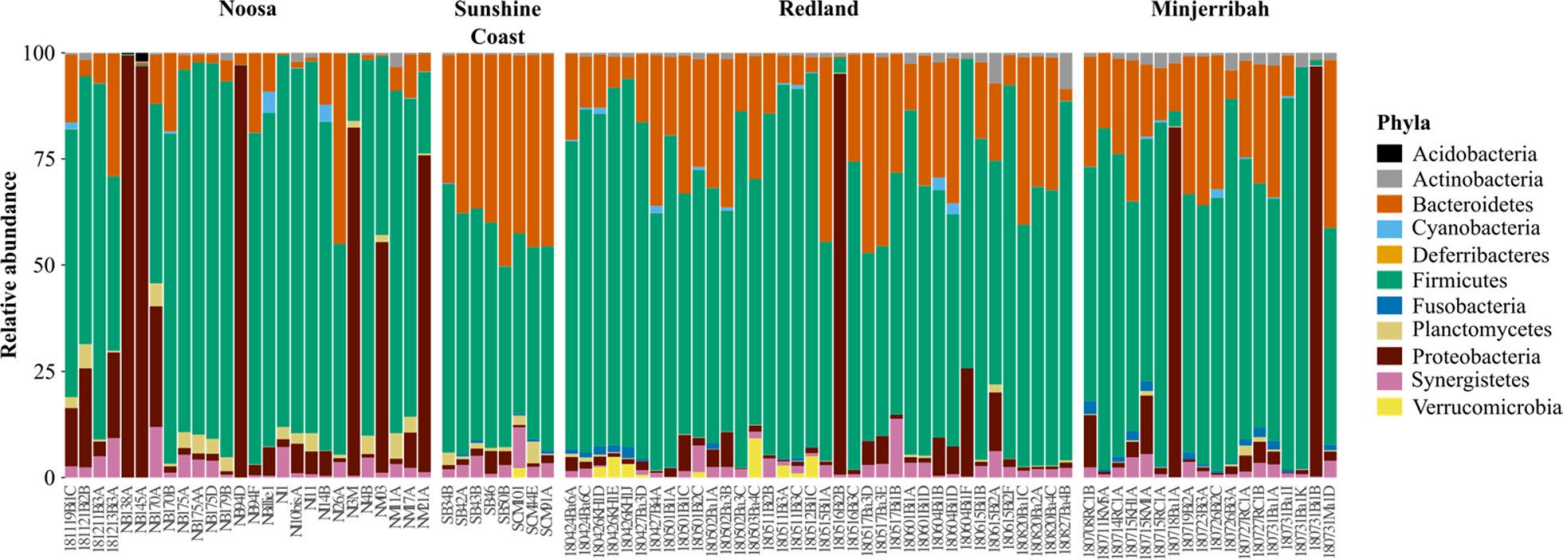
Relevant abundance of bacterial phyla for each individual koala (*n* = 88). The gut microbiota of koalas was predominated by *Bacteroides*, *Firmicutes* and *Proteobacteria*

We collected a total of 96 scat samples during large scale koala surveys conducted across four locations in South East Queensland (SEQ), Australia (Noosa_*n*_ = 26, Sunshine Coast_*n*_ = 11, Redland_*n*_ = 41, Minjerribah_*n*_ = 18). Following sequence/sample filtering and rarefaction, 2,579 gut microbial ASVs were identified across 88 koala samples (Table 1, Additional file 2: S2). In total, 11 phyla were identified across samples (Fig. 2), and in accordance with previous studies [28–30, 43], the gut microbiota of koalas across SEQ was dominated by the bacterial phyla *Firmicutes* (interindividual mean ± interindividual stdev; 62.54% ± 23.31%), *Bacteroidetes* (19.18% ± 14.74%) and *Proteobacteria* (12.51% ± 25.95%; Fig. 2, Additional file 2: S3). Of the 146 bacterial genera identified (Additional file 2: S3), the most abundant were *Ruminiclostridium 9* (16.10% ± 17.57%), *Bacteroides* (14.79% ± 12.94%), *Ruminococcus 1* (13.98% ± 13.17%), and *Shuttleworthia* (10.62% ± 13.69%). Across all 88 koalas, six ASVs were found in > 70% (83.14% ± 6.44%) of individuals (termed here the core microbiota). Four core ASVs were classified within the family *Ruminococcaceae* (found in 89.77–71.59% of all individuals), whilst the other two ASVs were classified within the families *Lachnospiraceae* (found in 830.68% of all individuals) and *Synergistaceae* (found in 87.50% of all individuals).

**Table 1:** Table denoting the number of samples used in each analysis

Location	Samples collected (pre-filtering)	Taxonomic composition of microbiota (post-filtering)	Landscape analyses			
			Location microbial diversity (after rarefaction)	Location host genetic differentiation (post-filtering)^†^	Pairwise mantel tests	
					M versus Geo distance	M versus G distance
Noosa	26	26	26	30	26	2
Sunshine Coast	11	8	8	12	8	3
Redland	41	36	36	30	36	25
Minjerribah	18	18	18	30	18	7
Total	96	88	88	102	88	37

M = microbial, Geo = geographic, G = genetic

^**†**^Additional koala DNA samples used for robust population genetic (F_st_) calculations

### Location significantly influences beta and alpha microbial diversity

**Fig. 3:**
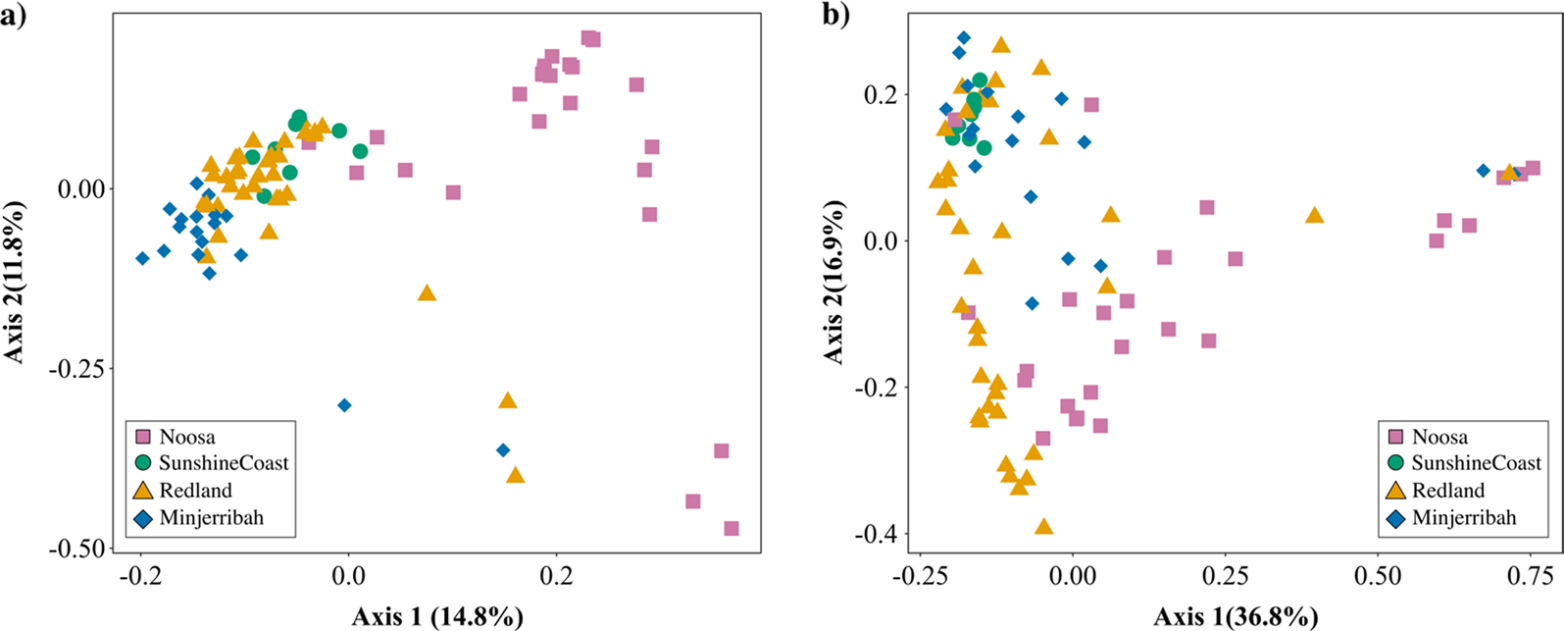
Location was found to significantly influence (*P* < 0.001) koala **a** gut bacterial community membership (unweighted UniFrac) and **b** gut bacterial community structure (weighted UniFrac) across South East Queensland, Australia. Significance between locations was determined using PERMANOVA tests. Each dot point represents the microbial profile of an individual koala (*n* = 88)

Location was found to significantly influence the gut bacterial membership (unweighted UniFrac: R^2^ = 0.213, *P* < 0.001; Fig. 3) and bacterial community structure (weighted UniFrac: R^2^ = 0.211, *P* < 0.001) of koalas, with significant differences found between all location pairs (determined using pairwise PERMANOVA tests; Additional file 2: S5). Year of sampling was not found to be significant (unweighted UniFrac and weighted Unifrac: R^2^ = 0.01, *P* > 0.05). Overall, whilst mean interindividual unweighted UniFrac distances were found to be, on average, larger between individuals from different locations (mean unweighted UniFrac distance = 0.58 ± 0.10), mean interindividual distances were also found to be relatively large between individuals from the same location (mean unweighted UniFrac distance = 0.48 ± 0.16).

**Fig. 4:**
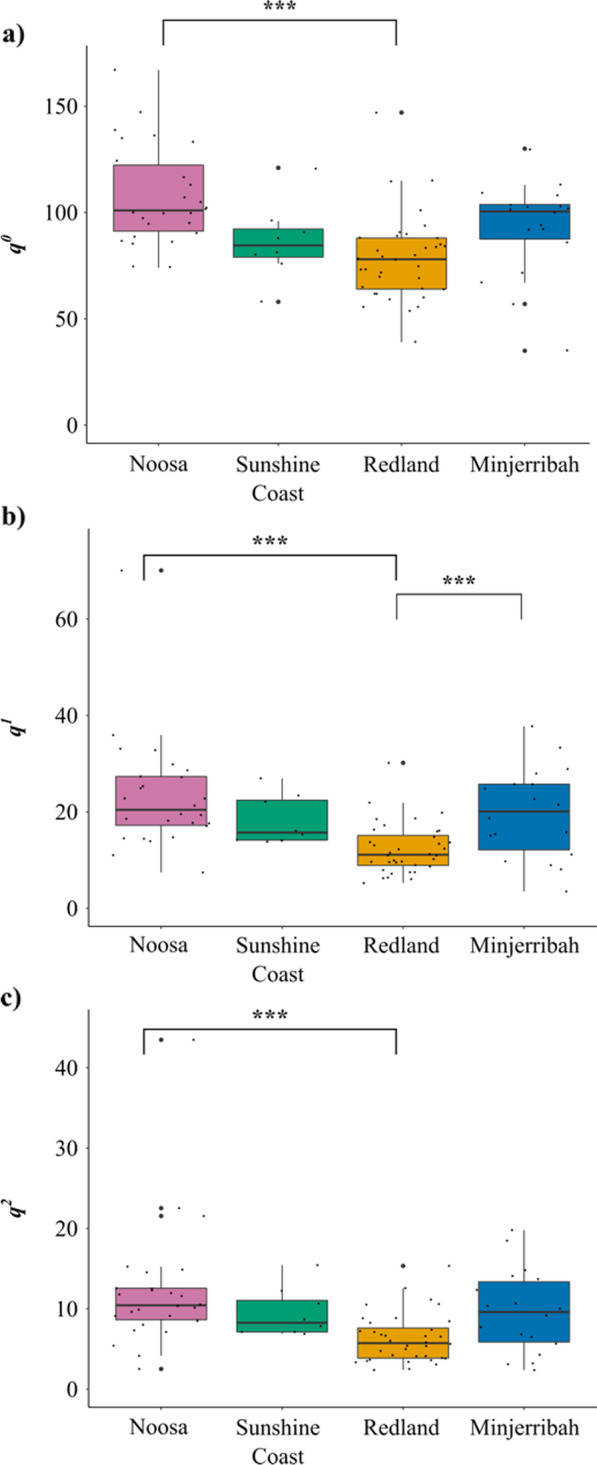
Location was found to significantly influence **a** microbial richness (*q*^0^), **b** number of typical (*q*^1^), and **c** te number of dominant (*q*^2^) bacterial taxa found in koala faecal samples (n = 88) across SEQ. Significant differences between pairwise locations were tested using Tukey HSD test or Nemenyi post-hoc test with significant differences represented with an asterisk(s) (*). Boxes show the median and the interquartile range, and whiskers represent 1.5 × interquartile range. Each dot represents the gut microbial diversity of an individual koala (*n* = 88)

We observed significant effect of location on gut microbial richness (*q*^0^; H_(3)_ = 31.14, *P* < 0.001), the number of typical microbial taxa (*q*^1^; *H*_(3)_ = 29.97, *P* < 0.001), and the number of dominant microbial taxa (*q*^2^; *H*_(3)_ = 21.53, *P* < 0.001), with Redland individuals showing lower alpha diversity compared to all other locations (significant pairwise comparisons are indicated in Fig. 4).

### Significant host genetic differentiation found between locations

**Fig. 5:**
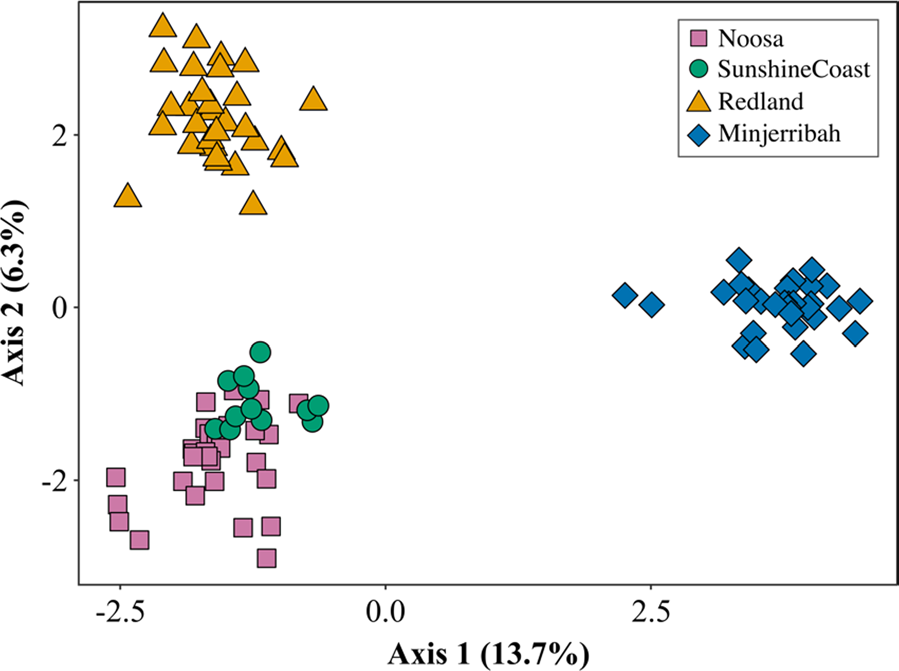
Location was found to significantly influence host genetic differentiation (F_st_ = 0.017–0.153, *P* < 0.05; Additional file 2: S5) across South East Queensland. Each dot point represents the genetic profile of an individual koala (*n* = 102)

**Fig. 6:**
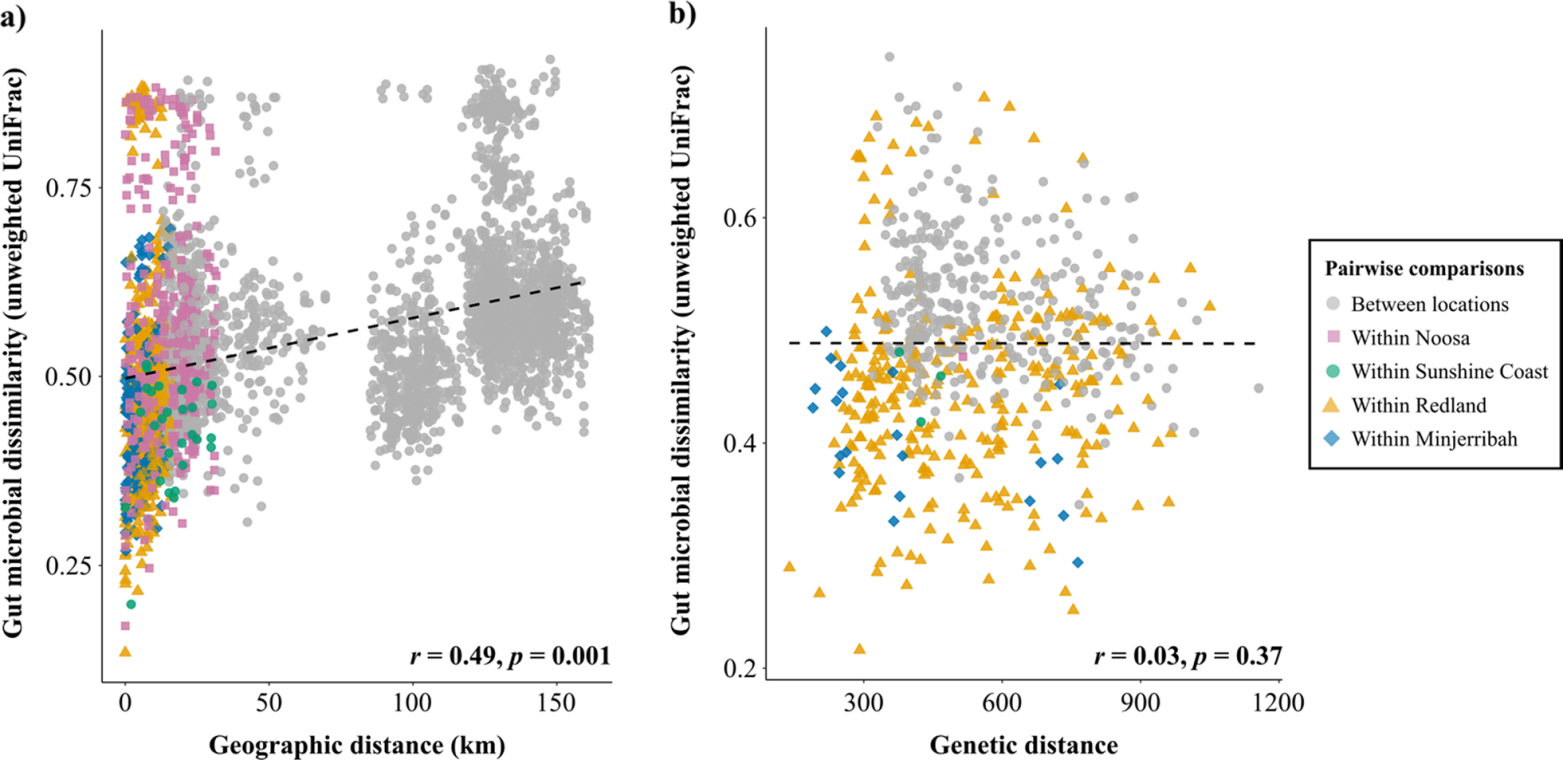
Pairwise dissimilarity between microbiome beta diversity (unweighted UniFrac) and **a** geographic distance and **b** genetic distance. Each point represents the pairwise distance between two individuals and are coloured depending on whether pairs came from the same location (coloured points) or different locations (grey points). The grey dashed line shows the overall trend; Mantel tests were run to determine correlation, where *r* is the correlation value and *P *value is the probability associated with *r*

We assessed whether host genetic trends mirrored koala microbial trends by comparing genetic and microbial differentiation patterns (Fig. 5). In order for meaningful pairwise F_st_ values to be calculated between locations, a larger genetic dataset was utilised to increase sample size and create even sampling across locations (*n*_*total*_ = 102; see Table 1 for sample sizes per location). There was significant host genetic differentiation between all locations (F_st_ = 0.017 – 0.153, *P* < 0.05; Fig. 6, Additional file 2: S5) with Redlands and Minjerribah showing the largest degree of host genetic differentiation (F_st_ = 0.153, *P* < 0.001; Fig. 6), and Noosa and Sunshine Coast showing the lowest degree (F_st_ = 0.017, *P* < 0.05; Fig. 6). Patterns of host genetic differentiation were found to differ to the microbial patterns found; whilst there was a low degree of host genetic differentiation between Noosa and Sunshine Coast (F_st_ = 0.017, *P* < 0.001) and Noosa and Redland (F_st_ = 0.066, *P* < 0.001), a relatively high microbial differentiation was found between these pairs of locations based on unweighted UniFrac and weighted UniFrac distances (Additional file 2: S5).

### Significant correlation between pairwise microbial diversity distances and geographic distance, but not genetic distance

To determine whether geographic and/or genetic distance between individuals correlated with pairwise microbial beta diversity measures across the landscape as a whole, we considered all pairwise comparisons of these measures regardless of location (i.e., samples were not categorised into locations). It should be noted that 88 samples were included in pairwise comparisons of microbial beta diversity and geographic distance, whilst 37 samples were included in pairwise comparisons of microbial beta diversity and genetic distance (Table 1, Fig. 1).

Pairwise geographic distance significantly correlated with gut microbial dissimilarity for both unweighted Unifrac (Fig. 6a) and weighted Unifrac (Additional file 1: Figure S3a). However, pairwise genetic distance did not significantly correlate with gut microbial dissimilarity for either unweighted Unifrac (Fig. 6b) or weighted Unifrac (Additional file 1: Figure S3b).

### Location influences the relative abundance of microbial taxa

**Fig. 7:**
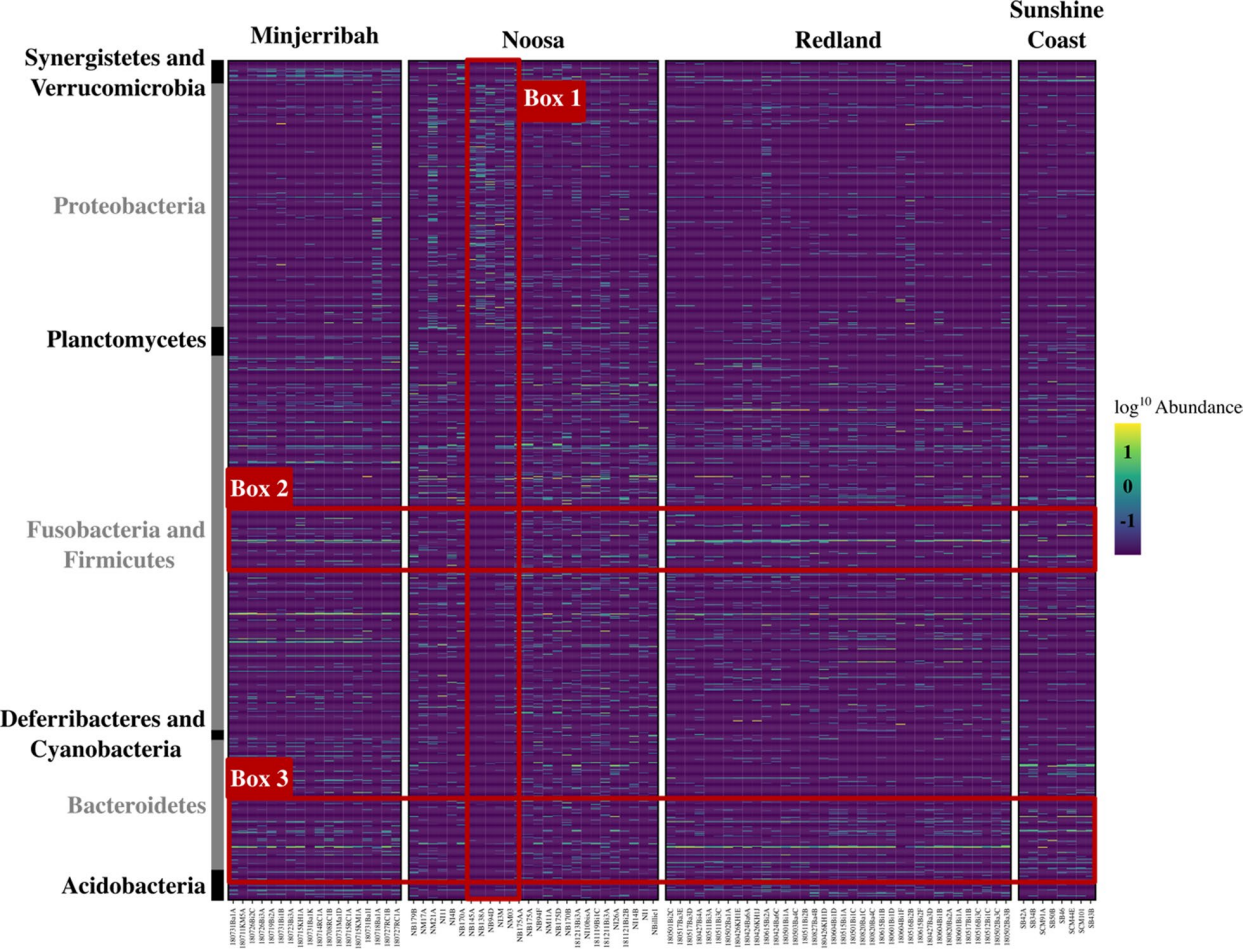
Relative abundance of koala gut microbial species across locations. Rows represent ASVs (*n* = 984), columns represent samples (*n* = 88), and the values in the heatmap represent logged relative microbial abundance, with green representing greater relative abundance. Black and grey shading in the left sidebar indicates the phylum level taxonomic assignment of each ASV (ASVs classified to 11 bacterial phyla). Box 1: five individuals within Noosa show an abundance of the phylum *Proteobacteria*, whilst showing a depletion in most other phyla. Box 2: ASVs taxonomically assigned to phylum *Firmicutes* are more abundantly represented in samples from Minjerribah, Redland and Sunshine Coast compared to samples from Noosa. Box 3: ASVs taxonomically assigned to the phylum *Bacteroidetes* were less abundantly represented in Noosa and Sunshine Coast samples

To ensure statistical robustness for differential abundance testing, we applied a stricter filtering regime where ASVs that only appeared in one sample and with a total sequence count of < 10 were removed (please see methods). Using a qualitative approach (clustering the relative abundance of all ASVs based on their phyla), two clusters of bacterial taxa were identified to have relatively low abundances in Noosa samples compared to all other locations. Cluster one (Fig. 7, Box 2) was made up of ASVs which belonged to the phylum *Firmicutes*, whilst cluster two (Fig. 7, Box 3) consisted of ASVs from bacterial phylum *Bacteroidetes*. Sunshine Coast samples also showed a relatively low abundance in the *Bacteroidetes* ASVs identified in Fig. 7, Box 3. In addition, five individuals from Noosa showed a considerable depletion in several bacterial phyla compared to all other individuals, whilst also showing an enrichment in ASVs belonging to the phyla *Proteobacteria* (Fig. 7, Box 1). When broken down into genera (Additional file 1: Figure S4), individuals from Noosa showed a largely reduced relative abundance in genera associated with the taxonomic orders *Gastranaerophilales* and *Bacteroidales*, and an enrichment in the genera *Tyzzerella 3, Rumuninococcacaea UCG-013*, *Rumuninococcacaea UCG-010* and *Rumuninococcacaea UCG-005*.

Using a quantitative approach, ANCOM returned 98 assigned ASVs with significant differences in their abundance across locations (Additional file 1: Figure S5a, Additional file 2: S6). Of these 98 ASVs, four ASVs were clear outliers in the ANCOM volcano plot with high values for both *W* (number of null hypotheses rejected) and *clr* (effect size). These ASVs were taxonomically classified as *Parabacteroides* (342c745aaab4586fdb7b7da23089b9b1; *W* = 981) which was highest in Redland, *Phascolarctobacterium* (3fe6c4b657a8178b42a5a439ee348cbf; *W* = 978) was found to be highest in Sunshine Coast, whilst *Intestinimonas* (33ff7d878cc110b97b821f6b5e1b7c29; *W* = 976) and *Synergistes* (3ee4a05ff2e318c68e0e148bb0e4390d; *W* = 969) were found to only occur in Sunshine Coast.

When ASVs were grouped at the genus level, ANCOM identified 28 genera with significant differences in their abundance between locations (Additional file 1: Figure S5b, Additional file 2: S7). Of these 28 genera, two genera were clear outliers in the ANCOM volcano plot, and were identified as belonging to genera *Marvinbryanti*a (*W* = 135) and *Synergistes* (*W* = 135). *Marvinbryanti*a was found to be at its highest abundance in Minjerribah and lowest abundance in Noosa, whilst *Synergistes* only occurred in Minjerribah.

## Discussion

In order to successfully conserve individuals, populations and species, Callicott et al. [13] outlined three principal conservation goals that have since become the core pillars guiding conservation research. Despite the growing recognition that the microbiome serves as an influential, albeit an often understudied, phenotypic trait, conservation strategies are often based primarily on landscape ecological surveys and, less extensively, on population genetic studies. Below, using the koala, we outline how microbiome research can contribute to each of the conservation goals outlined by Callicott et al. [13] and can support conservation initiatives and programs.

### Understanding natural variation in living systems

Incorporating metagenomics into conservation science allows us to understand not only the natural variation of a host across its natural range, but also its symbionts. Whilst limited studies have looked at gut microbial communities in the koala [17, 28–30, 43, 44], microbial variation has not been assessed across the larger landscape. Here, we found a large variation in the relative abundance of the dominant bacterial phyla *Firmicutes*, *Bacteroidetes* and *Proteobacteria* in the koala (Additional file 2: S3), which likely reflects the dietary differences between individuals. For example, Brice et al. [43] and Blyton et al. [17] found that the ratio of *Firmicutes* to *Bacteroides* significantly differed in their relative abundance depending on a koala’s diet. Whilst these conclusions are based on individuals with diets dominated by, or exclusively containing, a single species of *Eucalypt*; in the wild, koala diets are more likely to form a continuum [17], resulting in variation across the landscape of *Firmicutes* and *Bacteroides*. Furthermore, *Parabacteroides*, *Bacteroides* and *Ruminococcus* have previously been identified as dominating the bacterial community of koalas [29, 43], but here, we also found that genera *Ruminiclostridium* and *Shuttleworthia* dominate the gut bacterial communities of koalas ranging across South East Queensland.

Moreover, we found only six ASVs present in > 70% of all koalas, with all other bacterial taxa occurring in less than 66% of individuals. Interestingly, four of these ASVs were taxonomically assigned to the *Ruminococcaceae* family, one was assigned to the *Lachnospiraceae* family, and one was assigned to the *Synergistaceae* family. Members of the *Lachnospiraceae* and *Ruminococcaceae* families have previously been associated with a messmate diet [17] and have been identified as fibrolytic [47], whilst the *Synergistaceae* family is predicted to encode multiple pathways related to the degradation of toxic *Eucalyptus* plant secondary metabolites, therefore playing a key role in the koala’s ability to survive in a specialized dietary niche [28]. However, perhaps due to the individuality of a koala’s specialised diet, the lack of many ubiquitous ASVs across individuals suggests that multiple bacterial taxa may serve overlapping roles across the landscape, such that different bacterial populations can achieve the same function. Due to the koala’s highly specialised diet, the koala could be predicted to experience functional redundancy across microbial communities. Different bacterial taxa may aid in the detoxification of *Eucalyptus* plant secondary metabolites in the koala gut depending on diet, for example [28]. However, evidence for the commonality of functional redundancy in wild animal microbiomes is lacking. Therefore, as our understanding of the functional contributions of different microbiome members develops, we will increasingly be able to identify the sets of micro-organisms likely contributing to host health, and better determine which microbiome components should be prioritized in conservation decision-making [8].

### Understanding the composition, structure and function of living systems

In order to gain better insight into the composition, structure and function of living systems, we need to understand how host-microbial patterns shifts across time and space, as well as understanding what factors contribute to overall ‘landscape’ variation. Overall, our results suggest that the environment plays an important and key role in shaping the ‘landscape’ gut microbial profiles of koalas. First, we found that within-individual microbial diversity significantly differed between locations (Fig. 4), showing that individuals in particular locations have a significantly reduced or increased gut microbial diversity due to their environment. This result highlights the need to integrate microbial research into conservation biology, as changes to within-individual microbial diversity may be an early indication of changes in host health and fitness [48]. Second, we found extensive differences in gut microbial composition between individuals across the landscape (Fig. 3). Not only did beta diversity measures correlate with geographic distance (Fig. 6a), we also found a similarly strong effect of location on the gut microbial signatures of koalas (Fig. 3), even between neighbouring locations (for example, Noosa and Sunshine Coast). Finally, we also found large within-location variation in gut microbiota composition, indicating that gut microbial communities can differ across shorter geographic distances. This highlights that koalas display complex micro-spatial microbial variation as well as complex macro-spatial variation.

Baseline data on host-associated microbial communities allows us to uncover which symbiotic taxa are important for host health and fitness, as well as identify any occurrence of early gastrointestinal disease caused by a microbial imbalance (e.g. “dysbiosis”) caused by environmental disturbance [7]. For example, koalas from Noosa show a depletion in several ASVs associated with the *Bacteroidetes* and *Firmicutes* phyla, as well as the *Gastranaerophilales* and *Bacteroidales* orders (Figs. 4, 5). Of concern, both *Gastranaerophilales* and *Bacteroidales* have been shown to provide beneficial properties to their host [49, 50], suggesting that individuals from Noosa could be at greater risk of dysbiosis. Additionally, we found that the genus *Synergistes* only occurred in Minjerribah individuals. Whilst *Synergistes* has been suggested to play a key role in allowing the koala to subsist on *Eucalyptus* [28], it is not likely to be essential for all koala diets as it had low abundance in all other locations in this study and was not found be linked to disease in Brice et al. [43]. Instead, it may only be necessary for koalas feeding on certain diets in certain locations. Minjerribah, for example, is a sand island off the south east coast of Queensland and, therefore, likely differs in many environmental factors (vegetation, soil and leaf chemistry, for example). In addition, dietary analyses conducted by Melzer et al. [51] revealed that, compared to other mainland locations, forest red gum, blackbutt (*E. pilularis*) and scribbly gum (*E. racemosa*) were particularly prominent dietary components of Minjerribah koalas, with novel species such as *Allocasuarina* also occasionally eaten.

Whilst environmental factors can strongly affect the composition of the microbiota, the effect of population genetics is less clear [52]. The fixation index (F_ST_), a classic genetic measure which identifies population structure, is largely used when designing conservation strategies [7]. Similar to F_ST_, the gut microbiome can be driven by both nonadaptive population genetic processes, such as migration and drift, as well as differential selection pressures. By comparing population genetic differentiation to gut microbial differentiation, we are able to detect whether similar population patterns and selective pressures are at play in both. Whilst we found significant genetic and microbial differentiation between locations, these patterns of differentiation differed across the landscape in our study (Fig. 6, Additional file 1: Figure S3, Additional file 2: S5). For example, strong genetic differentiation was found to exist between Minjerribah and Redland, but the degree of gut microbial differentiation was much less pronounced (Additional file 2: S5). On the other hand, the opposite is true for Noosa and Sunshine Coast, where weaker genetic differentiation and extensive microbial differentiation were detected. This mismatch not only suggests that different evolutionary forces may be at play for a host’s microbiome and genetics, but also highlights the need for microbiota data to be considered when designing conservation strategies, otherwise important population structure may go undetected.

### Maintaining the resilience, and ability of living systems to persist, over time

In order to maintain and ensure the resilience and persistence of living systems over time, two metagenomic approaches need to be applied. First, spatial and temporal monitoring of host populations and their host-associated microbiota is essential. Microbial plasticity is the capacity of the microbial community of a host to change its composition (presence and/or absence, relative abundance) and/or gene-expression patterns (functionality) in response to physiological changes and variation in the external environment [2]. Whilst microbial plasticity has been shown to likely be an essential factor that facilitates host acclimation and adaptation [2], conservation science is yet to use metagenomic tools to identify landscape and populations trends, or early signs of microbial imbalance or dysbiosis.

Second, if an individual, population or species is under threat from environmental perturbations, intervention may be necessary (e.g., animal translocations or reintroductions). However, greater care needs to be taken regarding the specificity of host–microbe interactions during intervention [7], as mismatching may occur between an individual's microbiome and its new environment [53] or between native and introduced conspecifics [54] and could be detrimental, especially for specialist species. For example, mismatching during the translocation of koalas could result in the starvation of individuals if they are released into a new environment, face a sudden change in diet, and possess inappropriate gut microbes. Blyton et al. [17], for example, found that the gut microbiomes of wild-caught koalas were unresponsive to dietary changes when bought into captivity, highlighting the possible danger of dismissing gut microbial signatures when considering translocations. The administration of faecal inoculations has also been suggested to allow for the dietary expansion of species, which could be particularly important for specialist species. Whilst initial results of faecal inoculations in the koala suggest that it can assist in shifting their dietary intake [17], microbiota may behave differently in the intestinal tract of a recipient depending on how well the existing communities are established [55]. The extensive micro- and macro-spatial microbial variation of the koala may make ensuring the successful intake of new microbes difficult without relying on antibiotic treatment (which has been shown to lead to gastrointestinal dysbiosis [44]).

## Conclusions

Identifying the causal links between microbial perturbations, animal fitness and population level declines is not only essential to preserve a host and their associated microbial biodiversity, but it is also essential in maintaining biological diversity, ecological integrity and ecological health. Here, we show that the integration of metagenomic research into koala conservation practice can not only enrich our critical understanding of the ecology and evolution of this endangered native Australian marsupial, but this approach can also shed light on population structure that has, until now, gone undetected. We believe that successful conservation relies upon on the integration of ecology and metagenomics in order to protect species, their habitats, and ecosystems from excessive rates of extinction, and creating achievable conservation strategies and global change policies.

## Methods

### Study locations and sample collection

Large scale koala surveys were conducted between 2016 and 2018 by the Detection Dogs for Conservation team (University of the Sunshine Coast) across South East Queensland, Australia, with scats collected in locations defined in Fig. 1. Location boundaries are defined by local council borders in order to make this research valuable to council level conservation strategies.

Within each location, survey sites were located in conservation areas, recreational areas (e.g. urban parks), rehabilitation areas, wildlife corridors, National Parks and private properties. Koala scats yield the best DNA quality when they are fresh (determined to be less than one week old by identifying strong smell with a visible shiny mucus outer layer [56]). Scats were located using a non-invasive methodology where specially trained koala scat scent detection dogs [57] were led non-systematically and allowed to search freely without directions or constraints given by the handler, with surveys completed when the search was deemed to have covered the site extensively [58]. When the detection dog signalled that a koala scat was found, the dog handler visually confirmed the scat identification and recorded the location with a hand-held Garmin GPS (Alpha ® 100). Scats were collected in a sterile 50 ml centrifuge tube. In order to avoid direct human skin contact, potential contamination and the loss of koala DNA, the sterile tube and lid was used to collect several scats whilst avoiding the collection of ground matter. Samples were immediately put on ice during transportation and frozen at -20ºC until DNA extraction was conducted. In total, 96 scat samples were collected across the four sampling locations (Noosa (*n* = 26), Sunshine Coast (*n* = 11), Redland (*n* = 41) and Minjerribah (*n* = 18); Additional file 2: S4).

### Microbiota sample processing and analyses

#### Total genomic DNA extraction

For microbiota analyses, total genomic DNA was extracted from approximately 50–70 mg of material taken from the centre of each of the 96 scat samples using the QIAamp® PowerFecal® Pro DNA Kit (Qiagen) following the manufacturer’s protocol, with samples homogenised for seven minutes using a Vortex Adapter. Two extraction blank controls (EBCs) were included during total genomic DNA extraction.

#### Amplicon sequencing of scat samples

Total genomic DNA extractions were sent to and sequenced at the Australian Genome Research Facility (AGRF Ltd, Brisbane, Australia). All samples were sequenced at the same time and given anonymised acronyms (sample names did not contain location information) to eliminate technical bias. Both EBCs and one negative amplification control were included in the sequencing effort. Amplicon sequencing libraries were prepared for the Illumina MiSeq system using the 16S Metagenomic Sequencing Library Preparation guideline document, with all samples sequenced in a single Miseq run with lane number being randomised across samples. Briefly, a two-stage PCR process was used to amplify the primary product with an Illumina Nextera-adapter, with a secondary PCR to add the index on to the adapter. Two hypervariable regions (V3 and V4) of the 16S rRNA gene were amplified using the following primers: 341F (5′-CCTAYGGGRBGCASCAG-3′) and 806R (5′-GGACTACNNGGGTATCTAAT-3′). To discriminate individual koala samples after sequencing, both forward and reverse primers were labelled at the 5’ end with a combination of two different 8 bp tags (i.e. Nextera index strategy). The resulting 16S rRNA amplicons were measured by fluorometry (Invitrogen Picogreen), pooled at equimolar concentrations and sequenced on the MiSeq platform with the 300 bp paired end read chemistry.

#### Sequence assembly and quality control for gut microbiota

Sequence reads were filtered and processed using the DADA2 pipeline [59] in QIIME2 version 2019.4 [60]. We identified bacterial 16S rRNA sequence variants (Amplicon Sequence Variants; ASVs) using the SILVA 128 reference database [61]. Illumina sequencing generated a total of 3,970,881 reads (median of 36,040 reads per sample) and 4,439 ASVs after DADA2 processing. These sequences were further processed by removing non-bacterial ASVs (archaea, chloroplasts, and mitochondria), ASVs not assigned to the phylum taxonomic level, and ASVs with a total sequence count of < 2. Careful analysis of the negative control and the two EBCs was conducted in order to confirm that the gut microbial results and patterns reported here were not driven by contamination from these samples. A phylogeny was inferred for all ASV sequences with fasttree [62] based on a multiple sequence alignment generated by mafft [63]. Following filtering, all 88 samples were retained for subsequent analyses. These 88 samples contained > 4234 sequences (for sample rarefaction curves, see Additional file 1: Figure S2), where a total of 3,619,847 high quality genomic reads were generated and 4099 ASVs were identified (Additional file 2: S1).

#### Landscape analyses: assessing the impact of location on gut microbial profiles and diversity

To compare ASV diversity within (alpha diversity) and among (beta diversity) individuals, samples were rarefied to 4,200 sequences per sample (Additional file 1: Figure S2, Additional file 2: S2) to ensure a random subset of ASVs for all samples [64]. All alpha and beta diversity metrics were calculated in R [65].

Beta diversity was calculated between sample pairs using two metrics: unweighted UniFrac [66] and weighted UniFrac [67]. Unweighted UniFrac is an indicator of community membership (qualitative metrics), where only the presence and absence of ASVs are considered. Weighted UniFrac is a community structure metric (quantitative metrics), where the relative abundance of ASVs is taken into consideration. Both UniFrac metrics include information on the relative genetic relatedness of community members by incorporating phylogenetic distances between taxa [66–68].

To determine if location significantly influenced gut bacterial community membership (unweighted UniFrac) and structure (weighted UniFrac), statistical significance was assessed using PERMANOVA tests (permutational multivariate analysis of variance; [68] using the *adonis* function in the vegan package [69] in R. As samples were collected across multiple years in some locations, PERMANOVA tests included location and year-collected as fixed effects. Significance was calculated using R^2^ values, with 1000 permutations. In addition, pairwise PERMANOVA tests were run between locations using the *calc_pairwise_permanovas* function in the mctoolsr package in R (https://github.com/leffj/mctoolsr/).

To quantify within-sample diversity, we calculated Hill numbers [70] which weights taxa according to their abundance and is modulated with the parameter *q*. A *q* value of 0 is insensitive to ASV frequencies and yields a richness value, whilst a *q* value of 1 weights ASVs by their frequency, without disproportionately favouring either rare or abundant taxa [71] and is exactly the exponential of the Shannon index. A *q* value of 2 overweights ASVs and yields the multiplicative inverse of the Simpson index [72]. Hill exponents can be considered as the richness of all species (*q*^*0*^), the diversity of ‘‘typical’’ species (*q*^*1*^) or the diversity of dominant species (*q*^*2*^). Hill numbers were calculated per sample using the *vegan* package in R [73]. In order to determine if within-sample microbial diversity differed between locations, we ran an ANOVA (or Kruskal–Wallis non-parametric tests [74]) in R with location as the independent variable. Detailed differences in microbial diversity between locations were tested with *post-hoc* tests (Tukey HSD or Nemenyi posthoc test). All statistics were performed using the AOV.R or kruskal.test.R and the TukeyHSD.R or posthoc.kruskal.nemenyi.test.R function from the base package in R.

#### Detecting correlation between beta diversity distance and geographic distance

Mantel tests [75] were used to detect correlation between pairwise beta diversity distances (unweighted UniFrac, Binary Jaccard, weighted UniFrac and Bray Curtis coefficient) and pairwise geographic distance (Km) using the vegan package [76] in R, with 999 permutations for significance testing. Geographic distance was calculated between all pairs of individuals using genAlEx v6.5 [77]. All matrices used can be found in Additional file 2: S8-S11.

#### Landscape analyses: microbial differential abundance testing

To ensure statistical robustness for differential abundance testing, we applied a stricter filtering regime for the following analyses; in addition to the previously outlined filtering regime, ASVs that only appeared in one sample and with a total sequence count of < 10 were removed.

For qualitative assessment, the relative abundance of each ASV and genus were visualised for each location using heatmaps generated using the package qiime2R (version 0.99.22) in R. For heatmap visualisation, ASV counts were transformed to relative abundance, with abundance was log^10^ transformed after adding a pseudocount of 0.01% to each ASV (to better approximate a normal distribution of taxon relative abundance).

For quantitative assessment, we used analysis of composition of microbiomes (ANCOM) to test for significant differential abundance of ASVs and genera between locations. ANCOM calculates pairwise log-ratios (rather than using calculations based on proportions) between taxa to detect taxa that differ in their relative abundance between groups [78]. The *W* value represents the number of times the null hypothesis is rejected for a particular ASV, with centred logarithmic ratio (*clr*) transformations calculated to indicate effect size. To deal with zero counts, an arbitrary pseudocount value of one was added to each ASV prior to running ANCOM tests. ANCOM tests were completed on ASVs taxonomically assigned at either the genus or ASV level.

### Koala DNA sample processing

Koala DNA was isolated from scat samples which were collected as part of large-scale surveys conducted in 2016–2018 (Table 1).

#### Koala genomic DNA extraction

Koala DNA was isolated from intestinal epithelial cells on the surface of each scat by slicing off the outer-most layer of the scat using a scalpel. These surface slices were then used to extract koala DNA using the QIAamp DNA Stool Mini Kit (Qiagen), following an adapted version of the manufacturer’s protocol described in Schultz et al. [56].

#### Genotyping of individuals

Koala DNA was genotyped using a next-generation sequencing protocol for detecting Single Nucleotide Polymorphisms (SNPs) described in Kilian et al. [79] using probes (termed DArTcap) specifically designed for koalas. DArTcap is a targeted application of DArTseq™ technology [79] allowing for the sequencing of targeted markers and increased marker replication across samples.

#### SNP quality control filtering

For each individual sample, loci with an allele read coverage below five were assigned as missing data to reduce the amount of allelic dropout. Only samples with a call rate of at least 50% were retained. Loci quality was maximised by only retaining loci with more than 90% call rate across all samples and a minor allele frequency > 1%. Filtering was done in R using the R package dartR [80]. Duplicate samples were identified by calculating the number of mismatching loci between pairs of samples. Based on the level of mismatch between known duplicates and known parent–offspring genotypes, we determined that samples with 80% or more matching SNPs are likely to be duplicates. Of the identified duplicate samples, the sample with the least missing data was retained. After SNP filtering, 102 koala DNA samples with 479 SNPs were retained.

#### Host genetic differentiation

Genetic differentiation (F_st_) between all location pairs were calculated using the AMOVA function in genAlEx using 999 permutations.

#### Detecting correlation between beta diversity distance and genetic distance

In order to detect correlation between microbial beta diversity distance and genetic distance, the larger genetic dataset was subset to contain only those individuals which microbial data was available for (37 samples in total, Table 1). Individual-by-individual genetic distances [81] were calculated between all pairs of individuals using genAlEx. Mantel tests were used to detect correlation between pairwise beta diversity (unweighted UniFrac, Binary Jaccard, weighted UniFrac and Bray Curtis coefficient) and genetic distance using the vegan package in R, with 999 permutations for significance testing.

## Supplementary Information

**Additional file 1**. Supplementary figures.

**Additional file 2**. Supplementary sample metadata, OTU tables, and matrices.

## Data Availability

The dataset generated and analysed during the study are available under BioProject accession number PRJNA702467 from the National Center for Biotechnology Information (NCBI) sequence read archive (SRA).

## Abbreviations

16 S rRNA: 16 S ribosomal ribonucleic acid
ANCOM: Analysis of composition of microbiomes
ASV: Amplicon sequence variants
BP: Base pair
DNA: Deoxyribonucleic acid
EBCs: Extraction blank controls
PCoA: Principal coordinates analysis
PCR: Polymerase chain reaction
PERMANOVA: Permutational multivariate analysis of variance
SEQ: South East Queensland
SNP: Single nucleotide polymorphism
STDEV: Standard deviation
