# Supplementary material for: How microbiomes can help inform conservation: landscape characterisation of gut microbiota helps shed light on additional population structure in a specialist folivore
Source: Anim Microbiome. 2022 Jan 31;4:12. doi: 10.1186/s42523-021-00122-3 (PMC8802476; doi:10.1186/s42523-021-00122-3)
Supplement: Supplementary file 1 — Additional file 1. Supplementary figures. [file 42523_2021_122_MOESM1_ESM.docx]

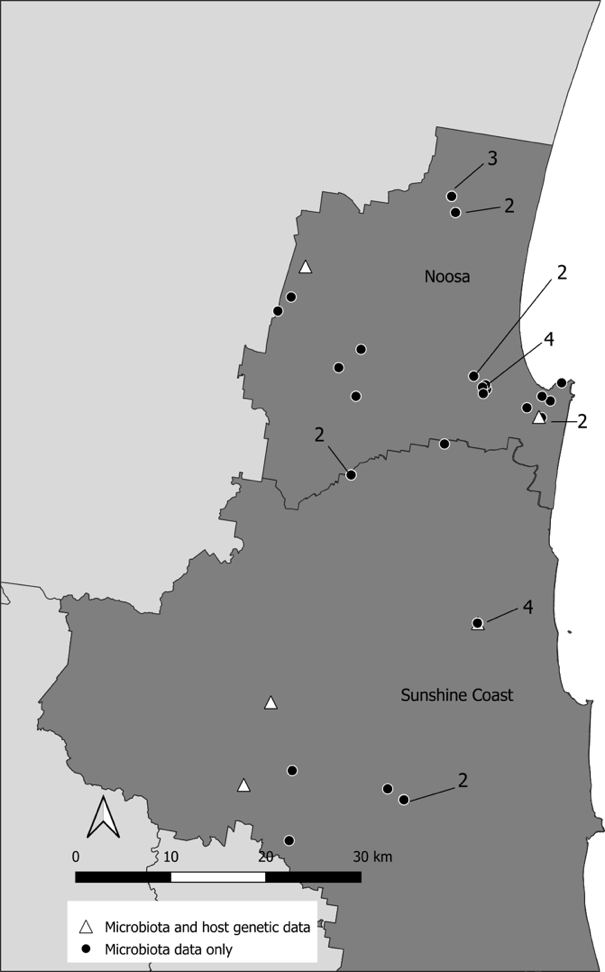


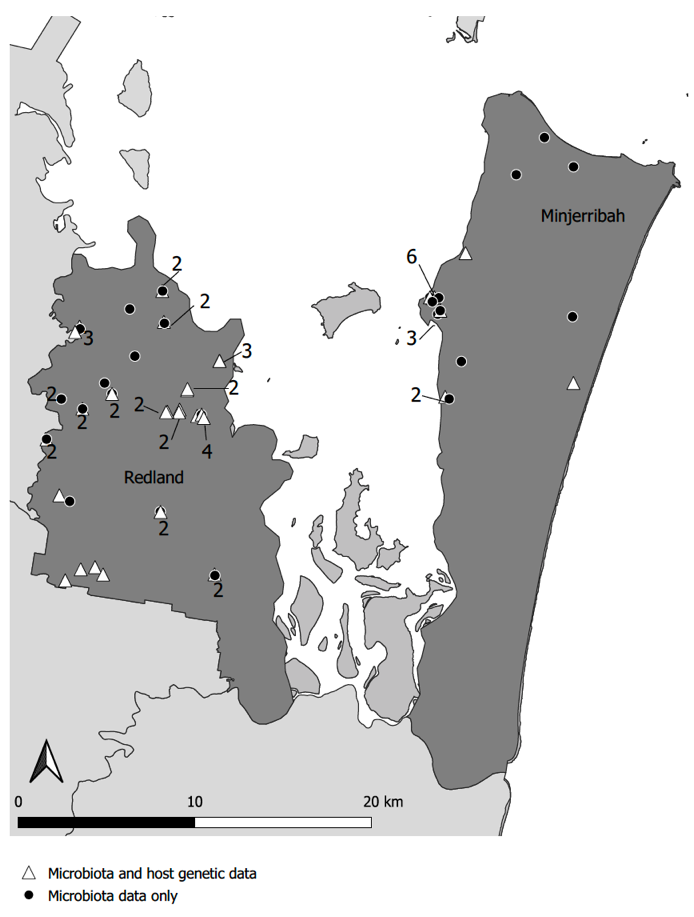


Supporting Information 1 Figure 1: Map showing where samples were collected across South East Queensland, highlighting the number of overlapping samples, and whether samples were used for metagenomic and/or genetic analyses.

Supporting Information 1 Figure 2: Rarefaction curves of species richness and sequencing depth for each koala sample per location. All samples were rarefied to 4,200 sequences per sample (black line in each plot) for alpha and beta diversity analyses.


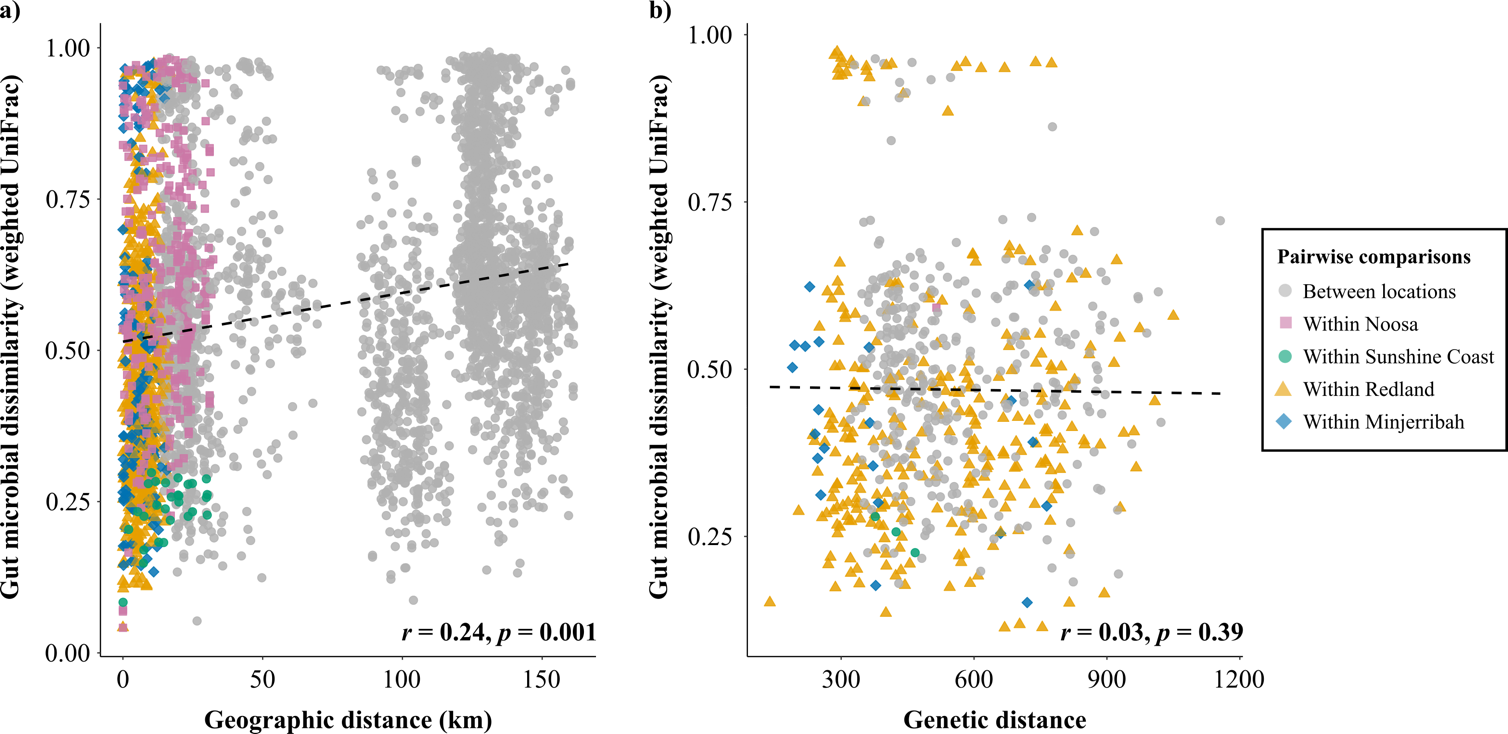


Supporting Information 1 Figure 3: Pairwise dissimilarity between koala gut microbial dissimilarity (weighted UniFrac) and (a) geographic distance and (b) genetic distance. Each point represents the pairwise distance between two individuals and are coloured depending on whether the comparison was within location (coloured points) or between locations (grey points). The grey dashed line shows the overall trend; Mantel tests were run to determine correlation where *r* is the correlation value and p-value is the probability associated with *r*.


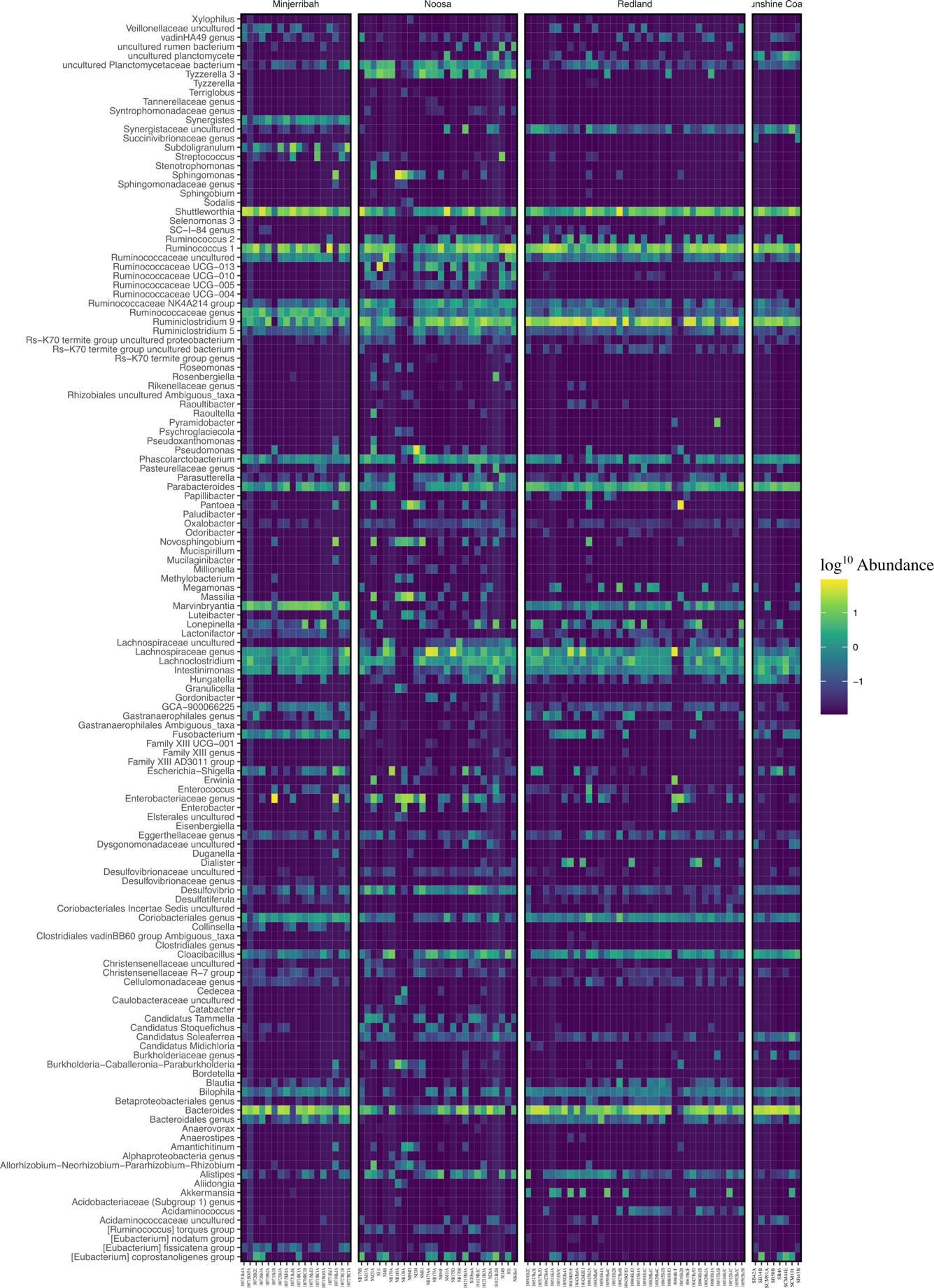


Supporting Information 1 Figure 4: Relative abundance of koala gut microbial genera across locations. Rows represent genera (*n* = 136), columns represent samples (*n* = 88), and the values in the heatmap represent logged relative microbial abundance, with green representing greater relative abundance


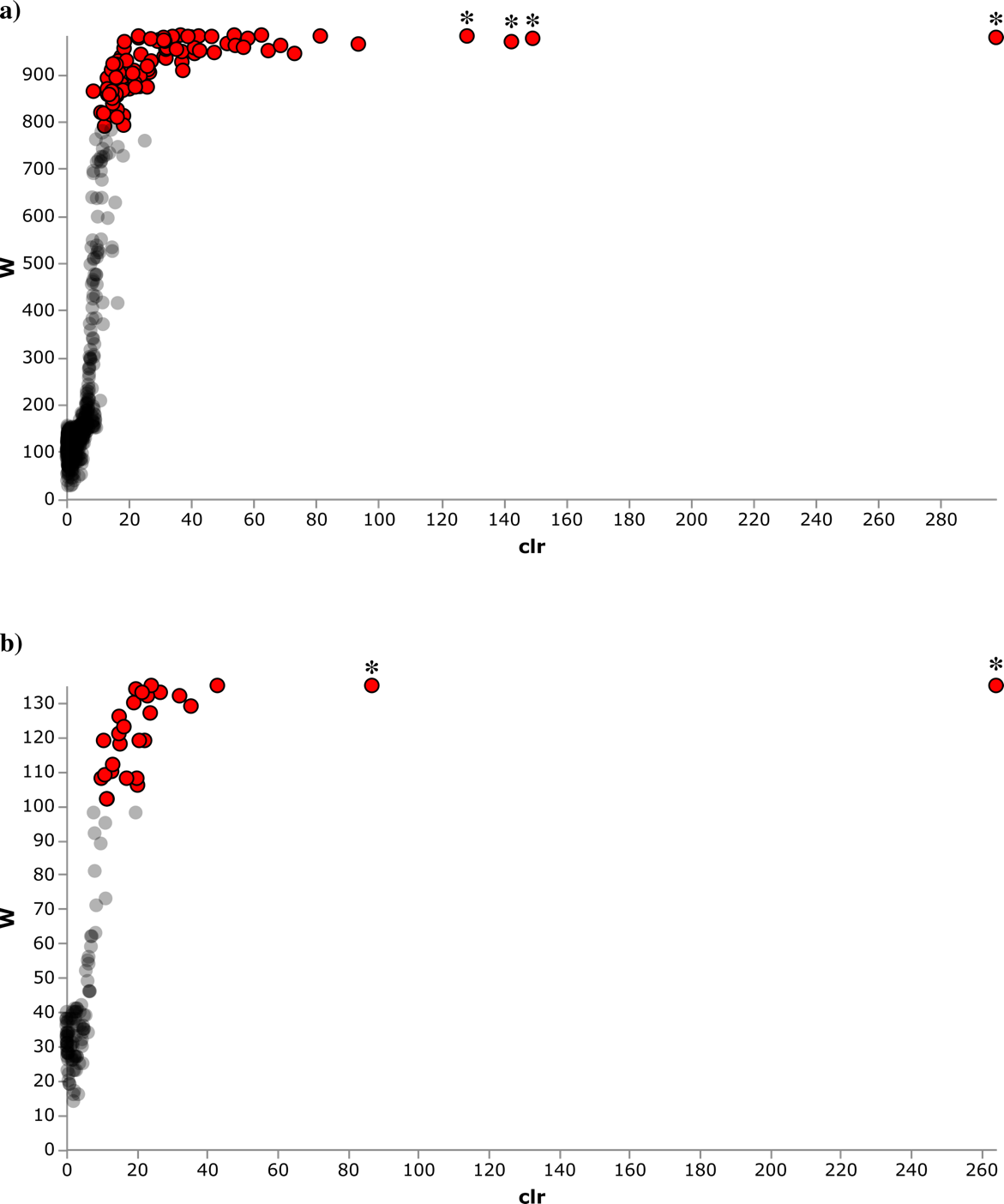


Supporting Information 1 Figure 5: Analysis of composition of microbes (ANCOM) volcano plot. Each point represents a) ASVs and b) genera, with those identified as having significant difference in abundance between locations highlighted in red. Points with asterisks (*) above indicate (a) ASVs or (b) genera that were identified as having high values for both *W* (number of null hypotheses rejected) and *clr* (effect size).
